# Development and validation of an online predictive model for biochemical recurrence after radical prostatectomy in elderly patients

**DOI:** 10.3389/fonc.2026.1753318

**Published:** 2026-03-16

**Authors:** Jie Liu, Hao Tan, Yang Lv, Bangxin Xiao, Xianglin Wu, Fang Wu, Mingzhao Xiao

**Affiliations:** 1 Department of Urology, The First Affiliated Hospital of Chongqing Medical University, Chongqing, China; 2 College of Artificial Intelligence Medicine, Chongqing Medical University, Chongqing, China; 3 Chongqing Cancer Multi-omics Big Data Application Engineering Research Center, Chongqing University Cancer Hospital, Chongqing, China; 4 School of Public Health, Chongqing Medical University, Chongqing, China; 5 Research Center for Medical and Social Development, Chongqing Medical University, Chongqing, China

**Keywords:** biochemical recurrence, predictive model, prognostic factors, prostate cancer, radical prostatectomy

## Abstract

**Objective:**

To develop and validate a novel model for predicting biochemical recurrence (BCR) in elderly prostate cancer (PCa) patients after radical prostatectomy (RP) and to create an accessible online tool for its clinical application.

**Methods:**

This retrospective study included patients who underwent RP at two independent medical centers. The initial cohort included 450 patients (2015-2022), which were randomly divided into a training set (n = 315) and an internal validation set (n = 135) at a 7:3 ratio. An independent cohort of 175 patients (2013-2023) was used as the external validation set. Potential predictors were screened via univariable Cox regression. The independent prognostic factors for BCR were subsequently identified via multivariate Cox regression. A predictive nomogram was developed on the basis of these independent factors. The model performance was assessed via time-dependent ROC curves, calibration curves, decision curve analysis (DCA), and Kaplan–Meier (KM) curves.

**Results:**

Cox multivariate regression analysis revealed that Gleason score (GS), lymph node metastasis (LNM), seminal vesicle invasion (SVI), and free prostate-specific antigen (fPSA) were independent risk factors for BCR after RP in the elderly population (all *P* < 0.05). The nomogram exhibited excellent time-dependent discriminative ability: the AUCs for 2-year, 3-year, and 5-year BCR-free survival were 0.857, 0.915, and 0.916, respectively, in the training set; 0.810, 0.846, and 0.856, respectively, in the internal validation set; and 0.698, 0.679, and 0.715, respectively, in the external validation set. Calibration curves demonstrated good agreement between the predicted BCR risk and actual incidence, and DCA confirmed that the model provides substantial clinical net benefit. We further developed an online tool (https://bcrnomapp.shinyapps.io/bcr-risk/) for personalized BCR-risk prediction.

**Conclusion:**

We developed a validated nomogram based on four independent risk factors—the Gleason score, lymph node metastasis, seminal vesicle invasion, and free PSA—for predicting BCR in elderly prostate cancer patients after radical prostatectomy. This model demonstrated robust predictive performance across multiple validation sets. The accompanying web-based tool facilitates rapid and individualized risk assessment, aiding in clinical decision-making.

## 1 Introduction

Prostate cancer (PCa) is one of the most prevalent malignancies of the male urinary system (1). Its incidence increases significantly with age, with over 1.46 million new cases and more than 396,000 deaths reported worldwide in 2022 (2). According to the Chinese Expert Consensus on the Diagnosis and Management of Prostate Cancer in the Elderly (2024 Edition), patients aged ≥ 65 years are defined as elderly prostate cancer patients (3). Notably, patients in this age group accounted for more than 60% of all PCa cases, making PCa a major cause of cancer-related mortality in elderly men (4). For patients with localized disease, radical prostatectomy (RP) represents a primary curative treatment option (5). However, biochemical recurrence (BCR) occurs in 20–50% of patients after RP (6, 7). The BCR serves as a critical indicator of disease progression and is strongly associated with an elevated risk of distant metastasis and cancer-specific mortality (8, 9). Therefore, the accurate identification of high-risk BCR populations and the development of reliable predictive models are crucial for guiding personalized postoperative follow-up, facilitating timely adjuvant therapy, and improving patient outcomes.

Current BCR prediction models (e.g., the Kattan nomogram and CAPRA-S score) are developed using data from all age groups and do not fully address the age-specific characteristics of elderly patients, including impaired physiological reserve, increased comorbidity burden, and potential disparities in tumor biology (10, 11). Moreover, most models are derived from single-center studies with inadequate external validation cohorts, restricting their generalizability across medical institutions. Additionally, the absence of user-friendly clinical translation tools (e.g., online calculators) impedes clinicians’ ability to meet practical demands for rapid postoperative risk assessment (12, 13).

In recent years, nomogram-based prediction models have been widely used to predict recurrence in urological malignancies (e.g., bladder cancer and renal cell carcinoma) because of their ability to integrate multidimensional prognostic factors and visually present individualized risk probabilities (14, 15). This study employed retrospective multicenter data from two institutions in Southwest China. Using univariable and multivariable Cox regression analyses, we aimed to identify independent prognostic factors for BCR after RP in elderly patients. We subsequently developed and validated a BCR-specific nomogram tailored to the elderly population. To enhance clinical utility, we created an accessible online tool, providing a practical resource for personalized postoperative management in this patient group.

## 2 Materials and methods

### 2.1 Subject selection

This cohort study retrospectively enrolled 450 patients aged ≥65 years with prostate cancer who underwent radical prostatectomy (RP) from the initial cohort (2015–2022). This cohort was randomly divided into a training set (n=315) and an internal validation set (n=135) at a 7:3 ratio. An independent external validation cohort consisting of 175 patients who met the same eligibility criteria (2013–2023) was used.

The inclusion criteria were as follows (1): preoperative diagnosis of PCa confirmed by transrectal prostate biopsy and (2) aged ≥65 years. The exclusion criteria were as follows (1): received preoperative neoadjuvant hormonal therapy (2); were lost to follow-up; and (3) had incomplete clinical or pathological data.

The ethic approval was obtained from the institutional review board and the patients’ informed consent was waived (K2024-193-01). This study was conducted in accordance with the Strengthening the Reporting of Observational Studies in Epidemiology (STROBE) guidelines.

### 2.2 Variable selection

A total of 21 variables were included in this study: age at diagnosis, body mass index (BMI), diabetes, hypertension, smoking history, alcohol consumption history, pathological T stage, Gleason score (GS), lymph node metastasis (LNM), bone metastasis, perineural invasion (PNI), positive surgical margins (PSM), seminal vesicle invasion (SVI), neutrophil–lymphocyte ratio (NLR), albumin–alkaline phosphatase ratio (AAPR), nutritional risk index (NRI), systemic immune–inflammation index (SII), free prostate–specific antigen (fPSA), the total prostate–specific antigen (tPSA), the free–total prostate–specific antigen ratio (f–tPSA), and lactate dehydrogenase (LDH). All laboratory indicators were defined as the most recent measurements obtained before surgery.

The following formulas were used for the relevant clinical and serological indicators:

BMI= Weight (kg)/[Height (m)]²; NRI = 1.487 × Albumin (g/L) + 41.7 × [preoperative body weight (kg)/ideal body weight (kg)], where ideal body weight (kg) = 22 × [height (m)]²; AAPR = Albumin (g/L)/Alkaline Phosphatase (U/L); NLR = Neutrophil count (10^9^/L)/lymphocyte count (10^9^/L); SII = Platelet count (10^9^/L) × NLR (16, 17).

According to the American Urological Association (AUA) guidelines, BCR was defined as at least two consecutive serum tPSA levels ≥0.2 ng/mL (18). The BCR-free survival time was calculated from the date of surgery to the date of BCR or the last follow-up.

### 2.3 Statistical analysis

Univariate and multivariate Cox regression analyses were performed to identify independent prognostic factors for BCR. The effect of each variable on BCR-free survival is presented as hazard ratios (HR) with 95% confidence intervals (CI). A prognostic nomogram was developed on the basis of the identified independent factors. The model’s discriminatory ability was assessed via a time-dependent receiver operating characteristic (ROC) curve (AUC). Calibration was evaluated with calibration curves, and clinical utility was determined by decision curve analysis (DCA). On the basis of the nomogram predictions, patients were stratified into low-risk and high-risk groups. Kaplan–Meier (KM) curves and log-rank tests were used to analyze and compare BCR-free survival between subgroups. Finally, an interactive web-based tool was developed via the Shiny package in R. All the statistical analyses were performed via Python (version 3.10.14) and R (version 4.3.2). P < 0.05 was considered statistically significant.

## 3 Results

### 3.1 Patient characteristics

The baseline clinical and pathological characteristics of the entire cohort are summarized in **Table 1**. A total of 625 patients who underwent RP were included in the final analysis. Among them, the initial cohort of 450 patients had a median follow-up of 51 months (IQR: 37–72). These 450 patients were randomly divided into a training set (n=315) and an internal validation set (n=135) at a 7:3 ratio. The external validation cohort consisted of 175 patients, with a median follow-up of 47 months (IQR: 35–70). The BCR rates were 25.4% (n=114) and 25.1% (n=44) in the initial cohort and external validation cohort, respectively. For the entire cohort, the median age was 72.0 years (IQR: 68.0–75.0), the BMI was 24.0 kg/m² (IQR: 22.0–26.0), and the tPSA level was 12.3 ng/mL (IQR: 2.8–33.2). Most patients (77.0%) had T1-T2 stage disease, and 67.5% had a GS ≤7.

**Table 1:** Baseline characteristics of patients in the total cohort, training set, internal validation set, and external validation set.

Variable	All patients (n=625)	Training set (n=315)	Internal validation set (n=135)	External validation set (n=175)
Age, years	72.0 (68.0-75.0)	72.0 (69.0-75.0)	71.0 (67.5-75.0)	71.0 (67.0-74.0)
BMI, kg/m²	24.0 (22.0-26.0)	24.0 (22.0-26.0)	24.0 (22.0-26.0)	24.0 (22.0-25.5)
NRI	108.0 (102.0-113.0)	109.0 (103.0-113.0)	109.0 (103.5-113.0)	105.0 (100.0-112.0)
AAPR	0.6 (0.5-0.7)	0.6 (0.5-0.8)	0.6 (0.5-0.7)	0.6 (0.5-0.7)
NLR	2.2 (1.6-2.9)	2.0 (1.6-2.7)	2.2 (1.6-2.6)	2.4 (1.7-3.3)
SII	368.9 (263.6-532.7)	345.6 (257.3-468.8)	376.2 (281.5-534.4)	426.6 (266.7-686.8)
LDH, U/L	167.0 (145.0-187.0)	161.0 (140.0-176.0)	165.0 (140.5-180.0)	180.0 (161.5-202.0)
f/tPSA	0.1 (0.1-0.2)	0.1 (0.1-0.1)	0.1 (0.1-0.1)	0.1 (0.1-0.2)
fPSA, ng/mL	1.4 (0.3-3.2)	1.4 (0.4-3.0)	1.6 (0.3-3.5)	1.1 (0.2-3.2)
tPSA, ng/mL	12.3 (2.8-33.2)	12.7 (3.5-33.4)	12.7 (2.2-38.0)	11.8 (2.3-25.0)
pT				
T1-T2	481 (77.0%)	238 (75.6%)	110 (81.5%)	133 (76.0%)
T3-T4	144 (23.0%)	77 (24.4%)	25 (18.5%)	42 (24.0%)
GS				
GS ≤ 7	422 (67.5%)	203 (64.4%)	91 (67.4%)	128 (73.1%)
GS>7	203 (32.5%)	112 (35.6%)	44 (32.6%)	47 (26.9%)
LNM				
Negative	558 (89.3%)	283 (89.8%)	123 (91.1%)	152 (86.9%)
Positive	67 (10.7%)	32 (10.2%)	12 (8.9%)	23 (13.1%)
Bone metastasis				
Negative	583 (93.3%)	300 (95.2%)	125 (92.6%)	158 (90.3%)
Positive	42 (6.7%)	15 (4.8%)	10 (7.4%)	17 (9.7%)
PNI				
Negative	343 (54.9%)	199 (63.2%)	69 (51.1%)	75 (42.9%)
Positive	282 (45.1%)	116 (36.8%)	66 (48.9%)	100 (57.1%)
PSM				
No	511 (81.8%)	267 (84.8%)	109 (80.7%)	135 (77.1%)
Yes	114 (18.2%)	48 (15.2%)	26 (19.3%)	40 (22.9%)
SVI				
No	550 (88.0%)	280 (88.9%)	122 (90.4%)	148 (84.6%)
Yes	75 (12.0%)	35 (11.1%)	13 (9.6%)	27 (15.4%)
Diabetes				
No	497 (79.5%)	258 (81.9%)	104 (77.0%)	135 (77.1%)
Yes	128 (20.5%)	57 (18.1%)	31 (23.0%)	40 (22.9%)
Hypertension				
No	356 (57.0%)	178 (56.5%)	77 (57.0%)	101 (57.7%)
Yes	269 (43.0%)	137 (43.5%)	58 (43.0%)	74 (42.3%)
Smoking history				
No	322 (51.5%)	160 (50.8%)	68 (50.4%)	94 (53.7%)
Yes	303 (48.5%)	155 (49.2%)	67 (49.6%)	81 (46.3%)
Alcohol drinking history				
No	369 (59.0%)	175 (55.6%)	87 (64.4%)	107 (61.1%)
Yes	256 (41.0%)	140 (44.4%)	48 (35.6%)	68 (38.9%)
BCR				
No	467 (74.7%)	239 (75.9%)	97 (71.9%)	131 (74.9%)
Yes	158 (25.3%)	76 (24.1%)	38 (28.1%)	44 (25.1%)
BCR-free survival, months	50.0 (36.0-72.0)	54.0 (38.0-75.5)	48.0 (36.5-67.5)	47.0 (35.0-70.0)

BMI, Body Mass Index; NRI, Neutrophil to Red Blood Cell Ratio; AAPR, Albumin to Alkaline Phosphatase Ratio; NLR, Neutrophil to Lymphocyte Ratio; SII, Systemic Inflammatory Index; LDH, Lactate Dehydrogenase; f/tPSA, Free to Total Prostate Specific Antigen Ratio; fPSA, Free Prostate Specific Antigen; tPSA, Total Prostate Specific Antigen; pT, pathological T stage; GS, Gleason score; LNM, Lymph Node Metastasis; PNI, Perineural Invasion; PSM, Positive Surgical Margin; SVI, Seminal Vesicle Invasion; ECE, Extraprostatic Extension; BCR, Biochemical Recurrence.

### 3.2 Univariate and multivariate cox analyses for prognosis

**Figure 1:**
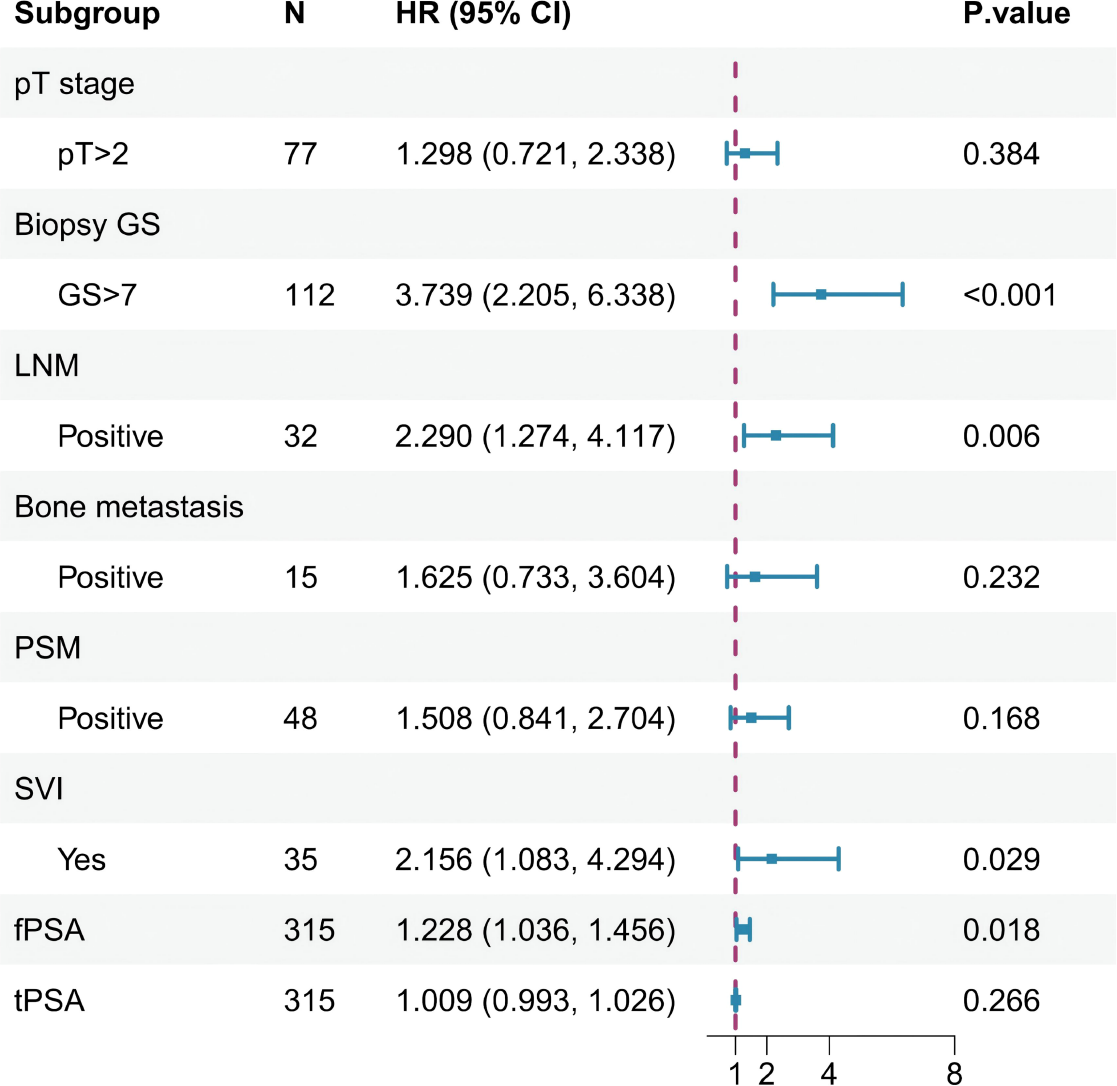
Forest plot showing the results of multivariate analysis for BCR-free survival.

Univariable Cox regression analysis was initially performed to screen all 21 candidate variables for their association with BCR-free survival. This analysis revealed eight variables significantly associated with BCR-free survival (*P* < 0.05): fPSA, tPSA, pathological T stage, pathological GS, LNM, bone metastasis, PSM, and SVI. These significant variables were subsequently entered into a multivariate Cox regression model. Four factors emerged as independent predictors for shorter BCR-free survival: GS >7 (HR = 3.74, 95% CI: 2.21–6.34, *P* < 0.001), LNM (HR = 2.29, 95% CI: 1.27–4.12, *P* = 0.006), SVI (HR = 2.16, 95% CI: 1.08–4.29, *P* = 0.03), and fPSA (HR = 1.23, 95% CI: 1.04–1.46, *P* = 0.02) (see **Table 2**, **Figure 1**).

**Table 2:** Univariate and multivariate Cox proportional hazards regression analyses for BCR-free survival.

Variable	Reference level	Univariate analysis		Multivariate analysis	
		HR (95% CI)	*P*-value	HR (95% CI)	*P*-value
Age	–	0.986 (0.935-1.040)	0.6	–	–
BMI (kg/m²)	–	1.017 (0.936-1.105)	0.69	–	–
NRI	–	1.007 (0.979-1.036)	0.63	–	–
AAPR	–	0.727 (0.178-2.968)	0.66	–	–
NLR	–	1.122 (0.870-1.448)	0.38	–	–
SII	–	1.000 (0.999-1.002)	0.41	–	–
LDH, U/L	–	1.000 (0.997-1.004)	0.8	–	–
f/tPSA	–	0.873 (0.052-14.568)	0.93	–	–
fPSA, ng/mL	–	1.434 (1.330-1.545)	**<0.001**	1.228 (1.036-1.456)	**0.02**
tPSA, ng/mL	–	1.033 (1.026-1.040)	**<0.001**	1.009 (0.993-1.026)	0.27
pT	T1-T2	2.953 (1.874-4.654)	**<0.001**	1.298 (0.721-2.338)	0.38
GS	GS ≤ 7	6.353 (3.842-10.506)	**<0.001**	3.739 (2.205-6.338)	**<0.001**
LNM	Negative	5.670 (3.435-9.358)	**<0.001**	2.290 (1.274-4.117)	**0.006**
Bone metastasis	Negative	3.011 (1.442-6.289)	**0.003**	1.625 (0.733-3.604)	0.23
PNI	Negative	1.460 (0.906-2.352)	0.12	–	–
PSM	Negative	2.151 (1.242-3.726)	**0.006**	1.508 (0.841-2.704)	0.17
SVI	No	3.559 (2.112-5.995)	**<0.001**	2.156 (1.083-4.294)	**0.03**
Diabetes	No	1.330 (0.753-2.351)	0.33	–	–
Hypertension	No	0.647 (0.400-1.047)	0.08	–	–
Smoking history	No	1.291 (0.819-2.036)	0.27	–	–
Alcohol drinking history	No	0.643 (0.401-1.030)	0.07	–	–

BMI, Body Mass Index; NRI, Neutrophil to Red Blood Cell Ratio; AAPR, Albumin to Alkaline Phosphatase Ratio; NLR, Neutrophil to Lymphocyte Ratio; SII, Systemic Inflammatory Index; LDH, Lactate Dehydrogenase; f/tPSA, Free to Total Prostate Specific Antigen Ratio; fPSA, Free Prostate Specific Antigen; tPSA, Total Prostate Specific Antigen; pT, pathological T stage; GS, Gleason score; LNM, Lymph Node Metastasis; PNI, Perineural Invasion; PSM, Positive Surgical Margin; SVI, Seminal Vesicle Invasion.

Bold values indicate statistically significant results (P < 0.05).

### 3.3 Prognostic nomogram model

**Figure 2:**
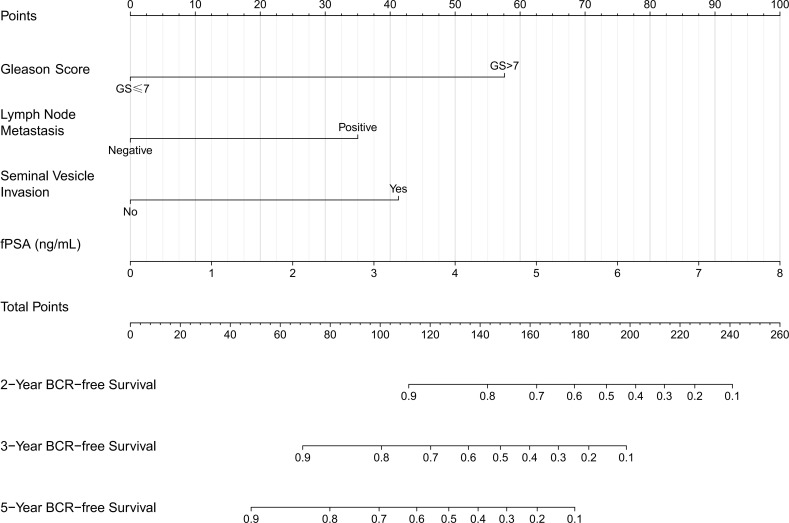
Nomogram for predicting 2-year, 3-year and 5-year BCR-free survival of older PCa patients after RP.

**Figure 3:**
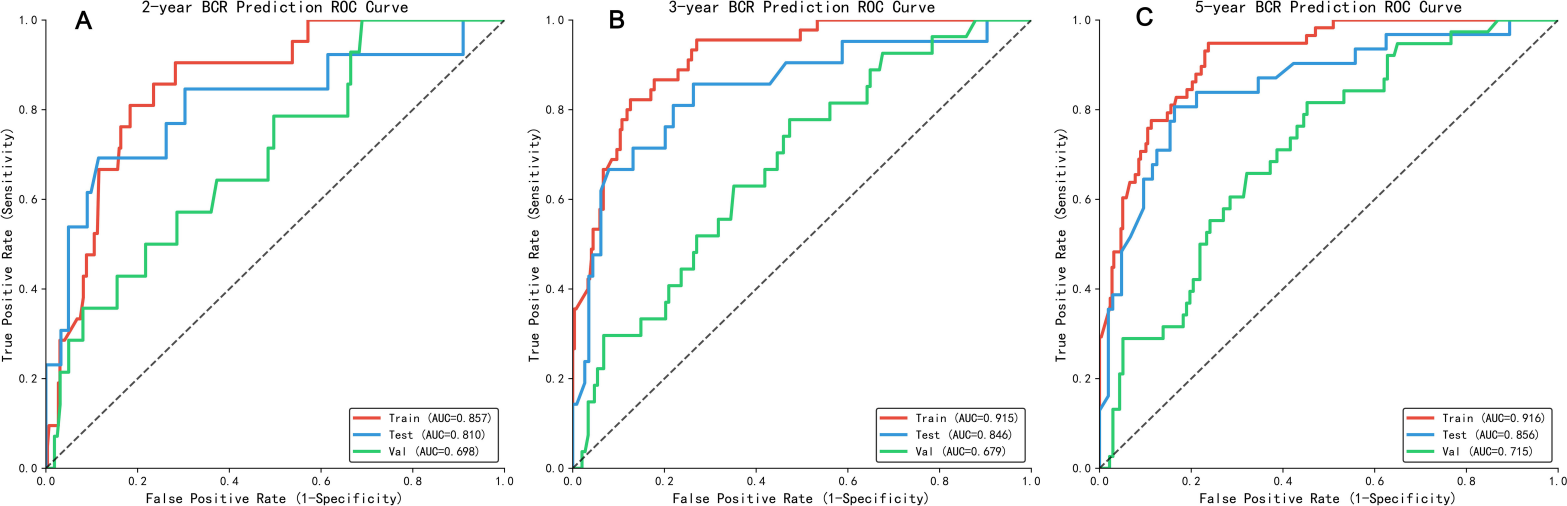
ROC curves for predicting 2-year **(A)**, 3-year **(B)**, and 5-year **(C)** BCR in the training set, internal validation set, and external validation set.

A nomogram was constructed based on all independent BCR-free survival related factors from the training set (as shown in **Figure 2**). Notably, free PSA (fPSA) is incorporated into the nomogram as a continuous variable—clinicians can directly input the measured preoperative fPSA value (ng/mL) to obtain the corresponding score (e.g., an fPSA of 2.5 ng/mL corresponds to ~30 points in the nomogram). The discriminative ability of the model was evaluated via time-dependent receiver operating characteristic (ROC) curves (**Figure 3**). For 2-year BCR-free survival prediction, the area under the curve (AUC) was 0.857 (95% CI: 0.786–0.928) in the training set, 0.810 (95% CI: 0.656–0.964) in the internal validation set, and 0.698 (95% CI: 0.558–0.839) in the external validation set, with the training set demonstrating excellent discriminative performance (AUC > 0.85) and the internal validation set showing good performance (AUC > 0.80). For 3-year prediction, the corresponding AUCs were 0.915 (95% CI: 0.876–0.955) (training set), 0.846 (95% CI: 0.742–0.949) (internal validation set), and 0.679 (95% CI: 0.573–0.784) (external validation set), with the training and internal validation sets maintaining robust discriminative ability. For 5-year prediction, the AUCs were 0.916 (95% CI: 0.880–0.951) in the training set, 0.856 (95% CI: 0.775–0.937) in the internal validation set, and 0.715 (95% CI: 0.628–0.802) in the external validation set, confirming excellent performance in the training and internal validation cohorts and acceptable discriminative ability in the independent external cohort.

**Figure 4:**
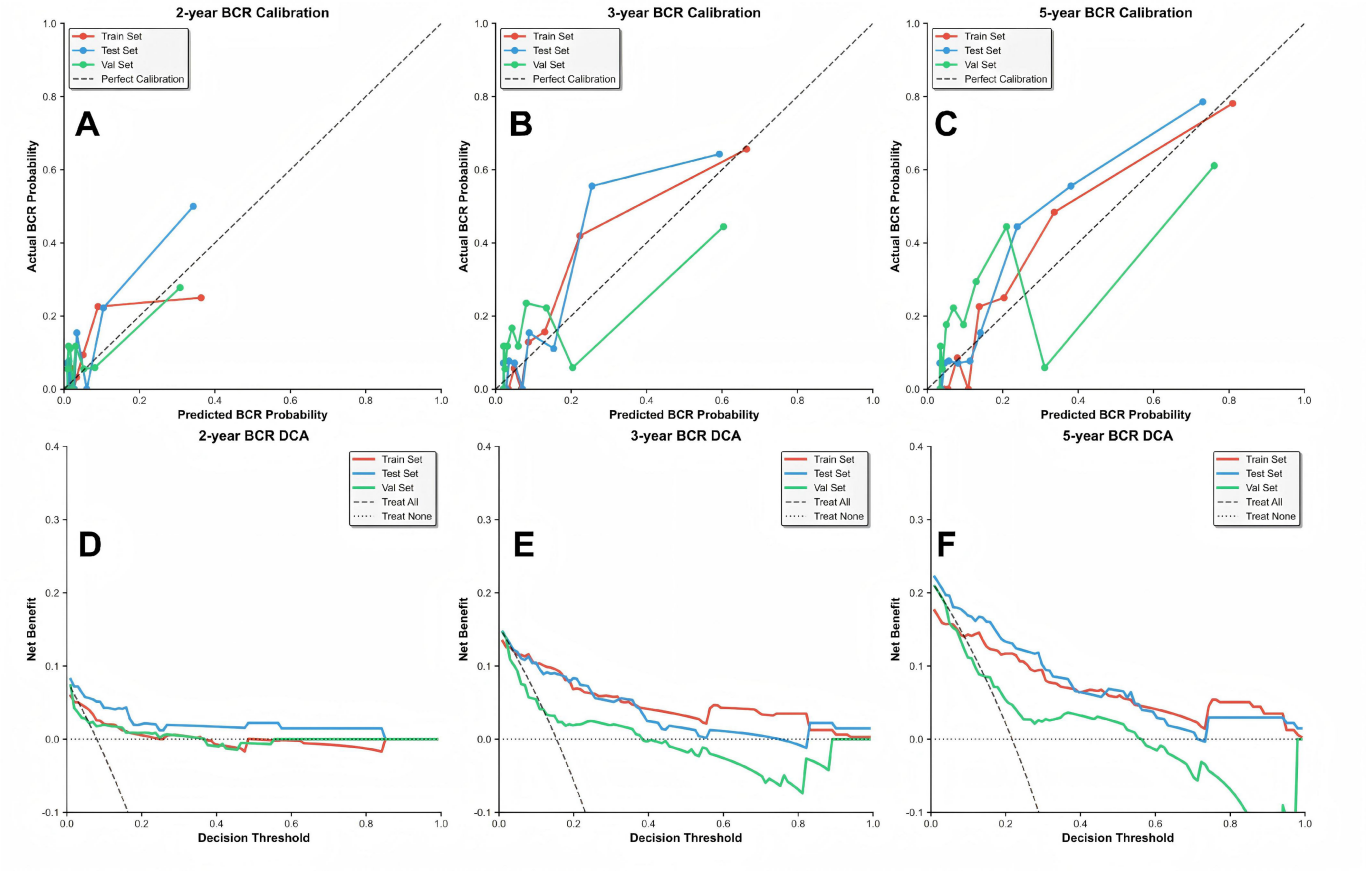
Calibration curves **(A–C)** and decision curves **(D–F)** of nomogram at 2-year, 3-year, and 5-year BCR-free survival in the training set, internal validation set, and external validation set.

The calibration curves revealed excellent agreement between the predicted probabilities and actual observed outcomes across all time points and datasets, indicating the model’s high reliability for individualized risk estimation. Furthermore, decision curve analysis demonstrated the substantial clinical net benefit of the nomogram across a wide range of threshold probabilities for predicting 2-, 3-, and 5-year BCR, outperforming the default strategies of treating all or no patients in both the internal and external validation sets (as shown in **Figure 4**).

### 3.4 Age-stratified performance of the prognostic model

To investigate the predictive heterogeneity of the developed model across distinct elderly subgroups, we stratified the entire cohort into three age strata (65–69 years, 70–74 years, and ≥75 years) and evaluated model performance within each stratum (**Table 3**). Across all age strata, the model retained stable discriminative ability (C-index >0.63; 5-year AUC >0.69) and favorable calibration (Brier score <0.23). These findings confirm that the model’s prognostic value is robust across different elderly subgroups.

**Table 3:** Performance of the prognostic model stratified by age groups in training, test and validation cohorts.

Age strata	Dataset	Sample size	C-index	AUC (5-year)	Brier score
65–69	Train	99	0.867	0.905	0.161
	Test	49	0.822	0.870	0.190
	Val	77	0.672	0.699	0.156
70–74	Train	122	0.891	0.925	0.075
	Test	50	0.746	0.803	0.089
	Val	57	0.631	0.704	0.224
≥75	Train	94	0.889	0.929	0.115
	Test	36	0.853	0.840	0.210
	Val	41	0.758	0.805	0.110
All Ages	Train	315	0.875	0.916	0.089
	Test	135	0.815	0.856	0.122
	Val	175	0.682	0.715	0.167

### 3.5 Comparison with CAPRA-S and Kattan postoperative model

**Figure 5:**
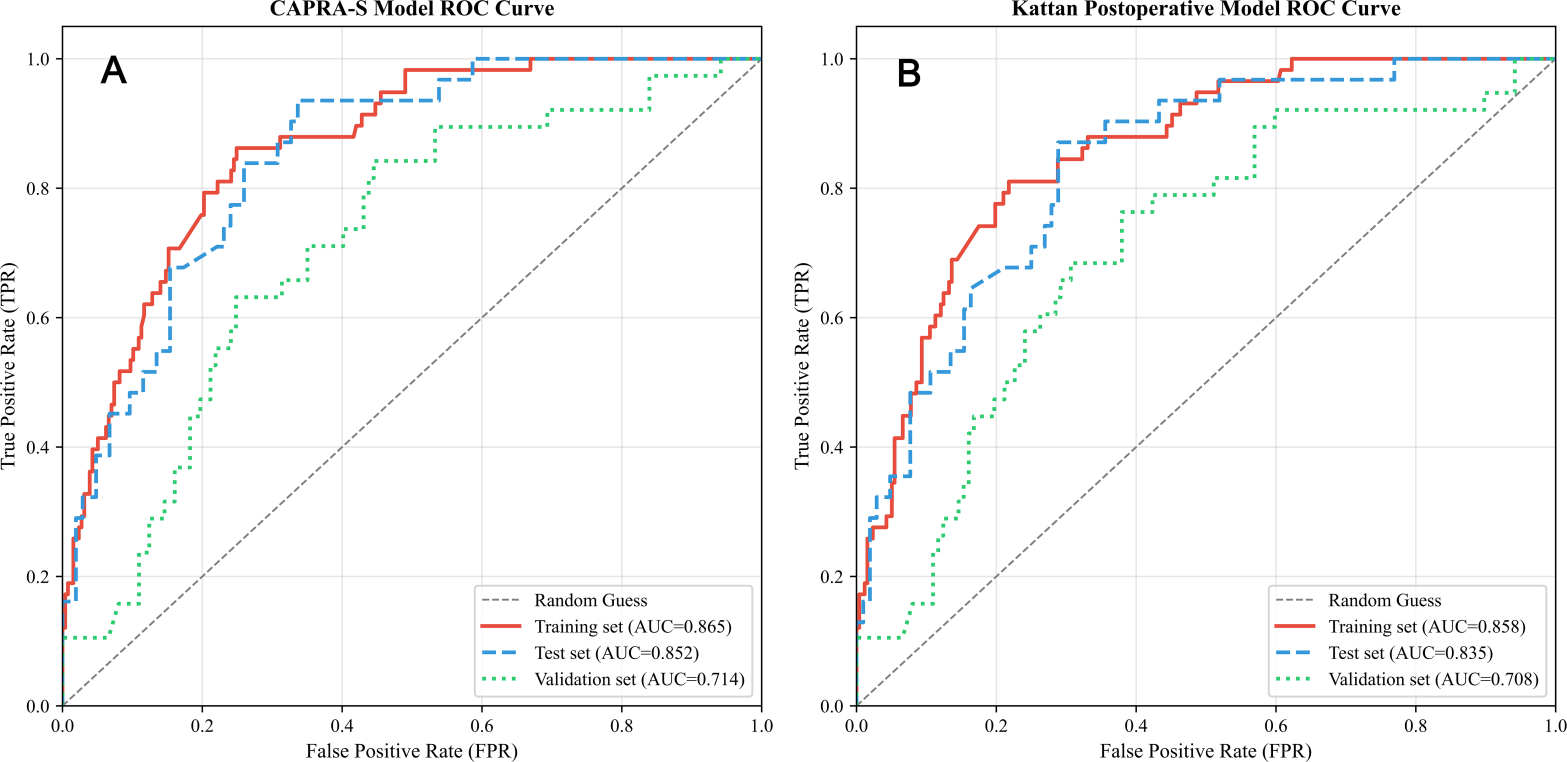
ROC curves of the CAPRA-S **(A)** and Kattan **(B)** Postoperative Models for Predicting 5-year BCR in the training set, internal validation set, and external validation set.

To evaluate the clinical competitiveness of our developed model, we compared its discriminative performance with two widely used prostate cancer BCR prognostic tools (CAPRA-S and Kattan postoperative model) in the same cohort (**Figure 5**). Our model exhibited superior discriminative ability across all datasets: in the training set, internal test set, and external validation set, the 5-year AUC values were 0.916, 0.856, and 0.715, respectively (**Figure 3C**). For the CAPRA-S model (**Figure 5A**), the corresponding 5-year AUC values were 0.865 (training set), 0.852 (test set), and 0.714 (validation set)—showing slightly lower performance than our model in the training and test sets. The Kattan postoperative model (**Figure 2B**) demonstrated 5-year AUC values of 0.858 (training set), 0.835 (test set), and 0.708 (validation set), with the weakest discriminative ability among the three models in the test and validation sets.

### 3.6 Risk stratification model

**Figure 6:**
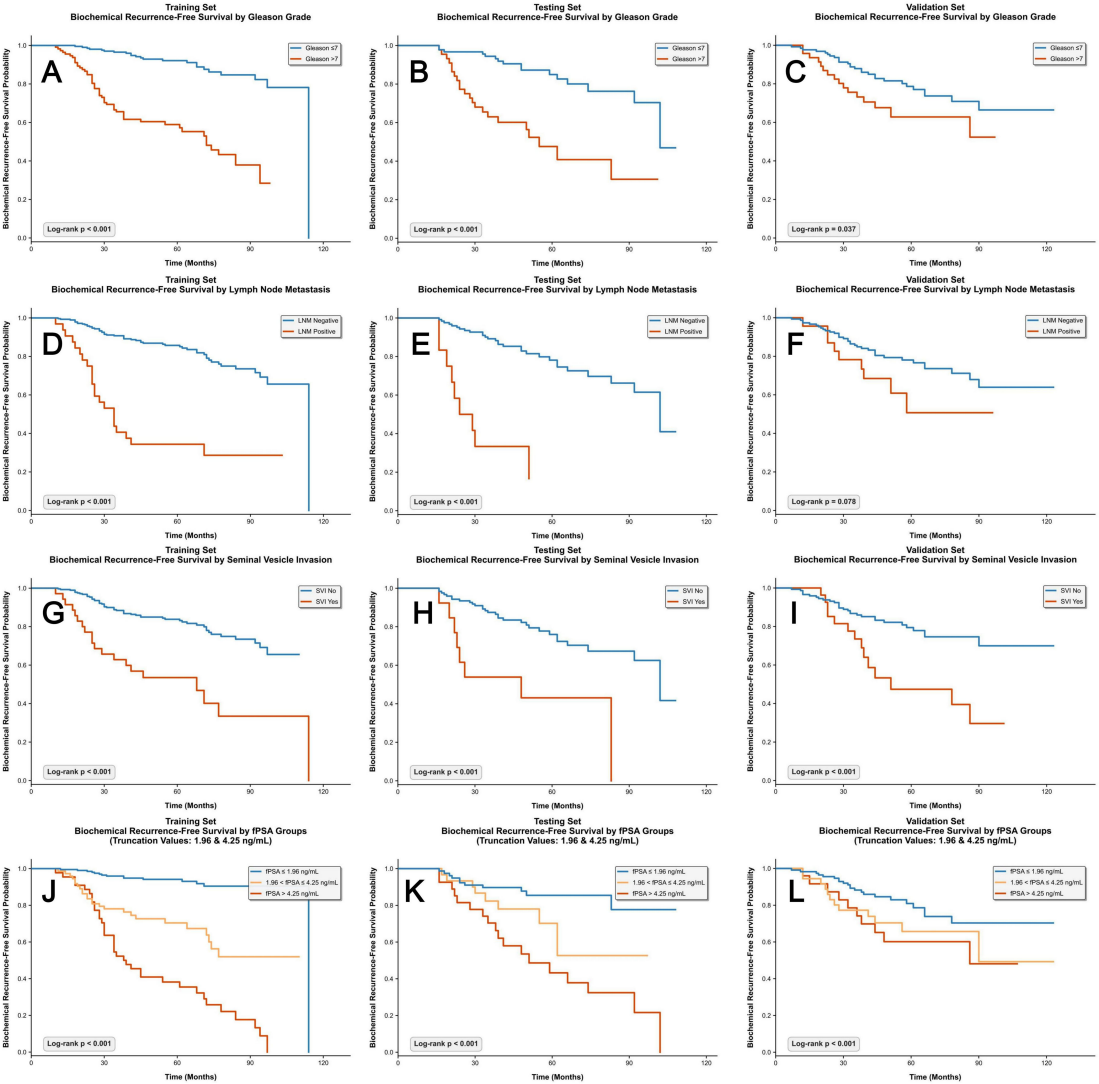
KM curves for BCR-free survival stratified by clinical-pathological factors in training, testing, and validation sets. **(A–C)** BCR-free survival stratified by Gleason score; **(D–F)** BCR-free survival stratified by lymph node metastasis; **(G–I)** BCR-free survival stratified by seminal vesicle invasion; **(J–L)** BCR-free survival stratified by fPSA groups.

The KM curves for each independent prognostic factor are presented in **Figure 6**. For fPSA, we further stratified patients into three subgroups (0–1.96 ng/mL, 1.96–4.25 ng/mL, ≥4.25 ng/mL) using X-tile software (**Figures 6J–L**). KM analysis showed statistically significant differences in BCR-free survival across these fPSA strata (p < 0.001): patients with fPSA ≥4.25 ng/mL had the shortest BCR-free survival, while those with fPSA <1.96 ng/mL had the longest, confirming that higher preoperative fPSA levels correlate with elevated BCR risk.

**Figure 7:**
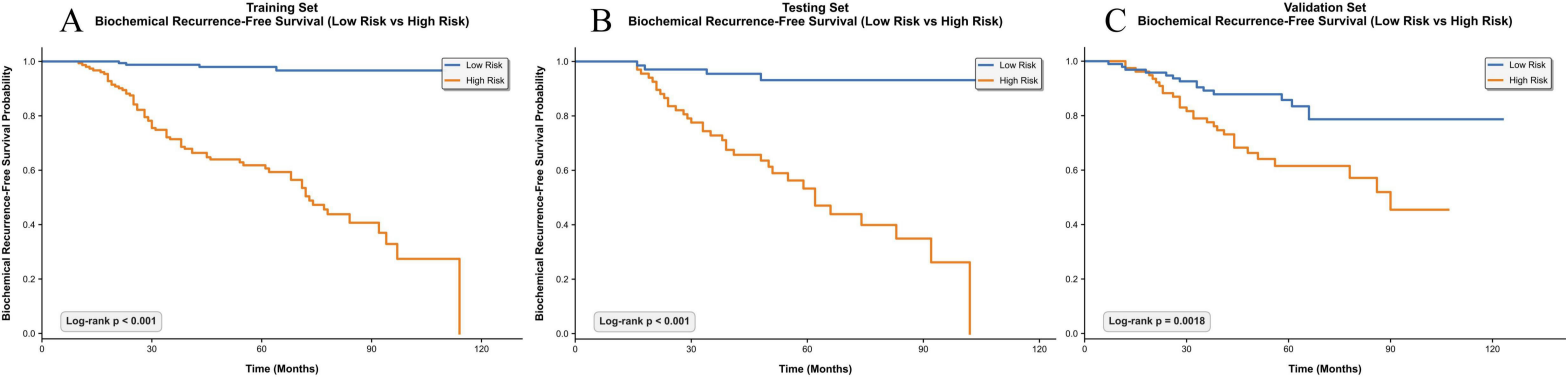
KM curves for BCR-free survival stratified by nomogram risk groups (low vs high risk) in the training set **(A)**, testing set **(B)**, and validation set **(C)**.

Patients were stratified into low-risk and high-risk groups according to the median total score of the constructed nomogram. KM analysis revealed statistically significant differences in BCR-free survival between the two groups, with the low-risk group exhibiting significantly longer BCR-free survival (*p* < 0.05; **Figure 7**). For high-risk patients, timely initiation of adjuvant prostate bed radiotherapy and intensive monthly PSA monitoring are recommended to enable early identification of biochemical recurrence and prompt salvage therapy, thereby optimizing their prognostic outcomes.

### 3.7 Online predictive tool

To increase the clinical accessibility of the nomogram for healthcare professionals, we developed an online tool (https://bcrnomapp.shinyapps.io/bcr-risk/) that provides nomogram-based services. Its automated calculation function generates personalized BCR-free survival probabilities for patients, and the scoring module supports early identification of high-risk individuals, thereby facilitating timely implementation of appropriate treatment and optimizing patient outcomes.

## 4 Discussion

In this multicenter study, we developed and validated a BCR prediction nomogram specifically for elderly patients after radical prostatectomy. This model, which integrates four readily available clinical parameters—GS, LNM, SVI, and fPSA—demonstrated robust and consistent predictive performance, calibration, and clinical net benefit across both the internal and external validation cohorts. By specifically addressing the distinct clinical profile of older adults, a population often underrepresented in existing all-age models, our tool provides a tailored and practical approach for postoperative risk stratification.

Our analysis confirms that GS, LNM, SVI, and fPSA are robust, independent predictors of BCR in the elderly RP population. This finding not only aligns with the established pathobiology of prostate cancer but also underscores the specific prognostic value of these factors in geriatric oncology.

The GS reaffirmed its status as the cornerstone for assessing tumor aggressiveness, emerging as the strongest contributor to our model. The substantial hazard ratio (HR = 3.74) we observed is consistent with the well-established prognostic value documented in previous literature (11, 19, 20). Prior studies have consistently identified a high GS and a PSA level >20 ng/mL as independent predictors of BCR for all patients (21, 22). Furthermore, the risk of BCR escalates with increasing GS. This gradient of risk is clearly illustrated by the 5-year BCR-free survival rates stratified by GS: 78.6% for GS = 6, 66.2% for GS = 7 (3 + 4), 51.1% for GS = 7 (4 + 3), and 35.5% for GS = 8-10 (23). In the context of elderly patients, where therapeutic decisions are frequently complicated by comorbidities and functional reserves, a high GS should be interpreted as a critical indicator for intensifying surveillance protocols, guiding the judicious use of adjuvant therapies, and framing patient-specific counseling rather than mandating uniform aggressive intervention.

LNM represents a definitive marker of systemic dissemination (24). This adverse pathological finding is strongly associated with an increased risk of BCR, as evidenced by Kang et al. (25), who demonstrated that lymph node invasion is frequently accompanied by a higher pathological stage and, consequently, a greater risk of BCR. This prognostic significance is further substantiated by a large-scale study of 2,608 patients undergoing RP, which confirmed LNM as an independent predictor of BCR (26). However, its clinical management in older adults presents unique challenges. Limited pelvic lymph node dissection, which is often performed to reduce surgical morbidity, may compromise the accuracy of staging. Furthermore, diminished tolerance to adjuvant radiotherapy or systemic therapy may limit effective treatment options after surgery. Consequently, for elderly patients preoperatively identified as high-risk for LNM, a meticulous evaluation of the potential survival benefit against the risks of more extensive surgery and subsequent treatments is paramount.

The SVI, a hallmark of locally advanced disease (pT3b) (27), was a powerful independent predictor in our model. This strong association with BCR reinforces the findings of prior research, which has further linked SVI to higher rates of metastasis and cancer-specific mortality (28). The critical relationship between SVI and adverse oncological outcomes after RP is further underscored by a clinical study of 4,486 RP patients by Park et al. (29). Their results demonstrated that the 5-year BCR-free survival rate for patients with SVI was merely 22.8%, significantly lower than the rates for those with extracapsular extension (62.0%) or positive surgical margins (52.4%) (P < 0.05), thereby establishing SVI as a dominant predictor of BCR. Given the recognized limitations of preoperative imaging in reliably detecting SVI, its positive identification on final pathology must be considered a nonnegotiable indication for instituting a more rigorous, long-term follow-up strategy.

The establishment of fPSA as an independent predictor of BCR, with a hazard ratio of 1.23 (95% CI: 1.04–1.46, P = 0.018), constitutes a pivotal feature of our model. We further validated its prognostic value using X-tile software to define optimal cutoff values, which categorized patients into low-, medium-, and high-risk fPSA groups. KM analysis confirmed that patients with high fPSA levels had significantly shorter BCR-free survival (**Figures 6J–L**), providing robust evidence for its discriminatory power. Biologically, fPSA is a monomeric glycoprotein predominantly expressed in prostate epithelial cells (30). Malignant PCa cells with high invasive and proliferative potential infiltrate prostate tissue, disrupting the blood-prostate barrier and enabling PSA leakage into the systemic circulation via capillaries and lymphatic vessels, thereby elevating fPSA levels (31). Concurrently, these aggressive tumor cells alter protease activity in the tumor microenvironment, impairing the binding of PSA to its endogenous inhibitors (e.g., α1-antichymotrypsin) and contributing to dysregulated elevation of preoperative circulating fPSA levels (32). Accordingly, preoperative fPSA levels not only reflect tumor-induced epithelial barrier disruption and dysregulated PSA release but also serve as a robust surrogate marker for tumor burden, invasive potential, and minimal residual disease. This finding is particularly salient for elderly individuals, a demographic with a high prevalence of benign prostatic hyperplasia (BPH), in whom tPSA levels are often confounded by non-malignant factors (33, 34). Our results resonate with the work of Goldberg et al. (35), whose study on PSA ratios in a prostate cancer population suggested that a free PSA ratio ≥0.10 following RP may be associated with a more aggressive tumor phenotype, implying a link between elevated fPSA and adverse postoperative outcomes. Furthermore, the incorporation of fPSA significantly enhances the accuracy of BCR prediction, a conclusion that aligns with the findings of Steuber et al. (36). Collectively, these observations demonstrate that fPSA offers a more tumor-specific reflection of oncological risk and provides crucial supplementary information for optimizing risk stratification in older patients.

Compared with established all-age models such as the Kattan nomogram and CAPRA-S score (10, 11), the innovations of our study are twofold: population specificity and biomarker optimization. We not only focused exclusively on the elderly population but also introduced fPSA, a variable with superior discriminatory potential in this cohort, thereby achieving a targeted enhancement of predictive accuracy. While the expected attenuation in the AUC was observed in the external validation set—a common phenomenon reflecting population heterogeneity—the model maintained acceptable discrimination. The lower AUC in the external validation set (0.679-0.715) compared with the training and internal validation sets may be related to the following inter-center differences: (1) Patient baseline characteristics: the proportion of GS>7 in the external validation cohort (26.9%) was lower than that in the training set (35.6%), and the positive rate of LNM (13.1%) was higher than that in the training set (10.2%). Tumor heterogeneity may affect model adaptability; (2) Surgical techniques: the difference in positive surgical margin rate (22.9% vs 15.2%) may lead to bias in the risk of BCR events; (3) Pathological evaluation standards: there were slight differences in the diagnostic thresholds for SVI among different centers, which may affect the consistency of prognostic factor determination. More importantly, its clinical value was unequivocally affirmed by decision curve analysis, which demonstrated a sustained net benefit across a wide range of risk thresholds, underscoring its practical utility.

Several limitations of our study warrant consideration. First, its retrospective design, while suitable for initial model development, carries an inherent risk of residual confounding. Prospective validation is needed to confirm our findings. Second, all the data were sourced from medical centers in Southwest China, which may affect the model’s generalizability to other ethnic and geographic populations. External validation in international cohorts is therefore a necessary next step. Third, the model’s predictive power could be refined by incorporating dynamic parameters such as PSA kinetics (e.g., PSA doubling time) and detailed comorbidity indices, which were not available for this analysis.

## 5 Conclusion

In summary, we developed and validated a BCR prediction nomogram specifically tailored for elderly prostate cancer patients. This model demonstrates robust performance and clinical utility, offering a quantitative tool for individualized postoperative management in this distinct population.

## Data Availability

The raw data supporting the conclusions of this article will be made available by the authors, without undue reservation.
